# Atomic-Scale Interfacial Magnetism in Fe/Graphene Heterojunction

**DOI:** 10.1038/srep11911

**Published:** 2015-07-06

**Authors:** W. Q. Liu, W. Y. Wang, J. J. Wang, F. Q. Wang, C. Lu, F. Jin, A. Zhang, Q. M. Zhang, G. van der Laan, Y. B. Xu, Q. X. Li, R. Zhang

**Affiliations:** 1 York-Nanjing Joint Centre (YNJC) for Spintronics and Nanoengineering, School of Electronics Science and Engineering, Nanjing University, Nanjing 210093, China; 2 Spintronics and Nanodevice Laboratory, Department of Electronics, University of York, York YO10 5DD, UK; 3 Hefei National Laboratory for Physical Sciences at the Microscale, University of Science and Technology of China, Hefei 230026, China; 4 Synergetic Innovation Center of Quantum Information and Quantum Physics, University of Science and Technology of China, Hefei, Anhui 230026, China; 5 Department of Physics, Renmin University of China, Beijing 100872, China; 6 Diamond Light Source, Didcot OX11 0DE, UK

## Abstract

Successful spin injection into graphene makes it a competitive contender in the race to become a key material for quantum computation, or the spin-operation-based data processing and sensing. Engineering ferromagnetic metal (FM)/graphene heterojunctions is one of the most promising avenues to realise it, however, their interface magnetism remains an open question up to this day. In any proposed FM/graphene spintronic devices, the best opportunity for spin transport could only be achieved where no magnetic dead layer exists at the FM/graphene interface. Here we present a comprehensive study of the epitaxial Fe/graphene interface by means of X-ray magnetic circular dichroism (XMCD) and density functional theory (DFT) calculations. The experiment has been performed using a specially designed FM_1_/FM_2_/graphene structure that to a large extent restores the realistic case of the proposed graphene-based transistors. We have quantitatively observed a reduced but still sizable magnetic moments of the epitaxial Fe ML on graphene, which is well resembled by simulations and can be attributed to the strong hybridization between the Fe 3*d*_*z2*_ and the C 2*p*_*z*_ orbitals and the *sp*-orbital-like behavior of the Fe 3*d* electrons due to the presence of graphene.

As a prototypical two-dimensional quantum system, graphene displays a combination of exceptional properties including large charge carrier mobility, high thermal conductivity, strong mechanical strength, excellent optical characteristics, electrically tuneable band gap, as well as the recently discovered long spin coherence length^1^,^2^,^3^,^4^. The revolutionary nature of graphene makes it a prime candidate to become a key material for the proposed spin transistors, in which the generation and tuning of spin-polarized currents are prerequisites^5^,^6^,^7^. In pristine state, graphene exhibits no signs of conventional spin-polarization and so far no experimental signature shows a ferromagnetic phase of graphene. This gap is now filling up by combined efforts in multi-disciplinary research. The FM/graphene heterojunction is one of the most promising avenues to realise efficient spin injection into graphene^8^,^9^,^10^,^11^,^12^,^13^,^14^. Perfect spin filtering for interfaces of graphite and Ni or Co has been predicted, which is insensitive to interface roughness due to the intrinsically ordered nature of graphite^10^. Fascinating properties of spin transport phenomena were presented in the Co/graphene system^11^,^12^, though theoretical calculations show that the atomic magnetic moment of Co can be reduced by more than 50% when absorbed on graphene surface^13^. An inserted graphene sheet can drastically improve the spin-injection efficiency from the FM into silicon^14^.

In any proposed graphene-based transistors, the best opportunity for spin transport could only be achieved when no magnetic dead layer exists at the FM/graphene interface. Previous studies on various FM/semiconductor (FM/SC) heterojunctions revealed the possibility that the magnetic ordering near a region of the surface or interface of FM/SC may be modified due to interdiffusion, termination and hybridization; and controversial reports make this issue rather complex^15^,^16^,^17^,^18^,^19^,^20^,^21^. Calculations for transition-metal/nanotube hybrid structures exhibit substantial magnetism^17^. For Fe-, Co-, and Ni-doped carbon nanotubes, the interactions are found ferromagnetic for Fe and Co while nonmagnetic for Ni^18^. A ~1.2 nm magnetic dead layer of Co was observed on a topological insulator surface^19^. Whether a deposited FM on graphene is magnetically ordered at the FM/graphene interface is a must-addressed issue before an efficient graphene-based transistor can be developed. In this Letter, we present a comprehensive XMCD study of the ML epitaxial Fe/graphene interface, combined with DFT calculations. The experiments have been performed using a specially designed FM_1_/FM_2_/SC structure that to a large extent simulates the realistic FM/graphene interface of the proposed graphene-based transistors^4^,^5^,^6^ and at the same time allows a direct determination of the interface magnetism of FM/graphene.

Fundamentally all the intriguing spintronic phenomena observed in the FM/graphene heterojunctions strongly depend upon the interfacial hybridization and magnetic exchange interaction^8^,^9^,^10^,^11^,^12^,^13^,^14^. A direct demonstration of the magnetic and electronic state of the FM/graphene interface down to ML scale remains a nontrivial task, even today, partially due to the inaccessibility of the buried layer between the topmost atoms and substrate. On the one hand, for samples comprising of several nanometers thick FM atop the SC substrate, it is always hard to separate the contributions of the interface and the bulk magnetization. On the other hand, the low coverage of FM (in the form of atoms and clusters) will be paramagnetic or ferromagnetic with extremely low Curie temperatures (*T*_*c*_)^22^,^23^, and consequently no longer be representative of a realistic device. Moreover, while the FM atoms reduce to a minute amount, many global detection techniques become invalid, as the experimental conditions like vacuum, sensitivity, cryogen etc. must be simultaneously satisfied at a high level. To overcome these obstacles, we employed a unique FM_1_/FM_2_/SC structure (in this letter, FM_1_ = 30 MLs Ni, FM_2_ = 1 ML Fe, and SC = graphene). The thick FM_1_ layer provides the FM_2_ ML with a source of exchange interaction and these two together restore the interfacial behavior of the thick ferromagnetic FM_2_/SC. Combined with the unique elemental selectivity of XMCD, such structure allows direct observation of the magnetization of FM_2_ at the FM_2_/SC interface.

## Results and discussions

### XMCD measurements

**Figure 1:**
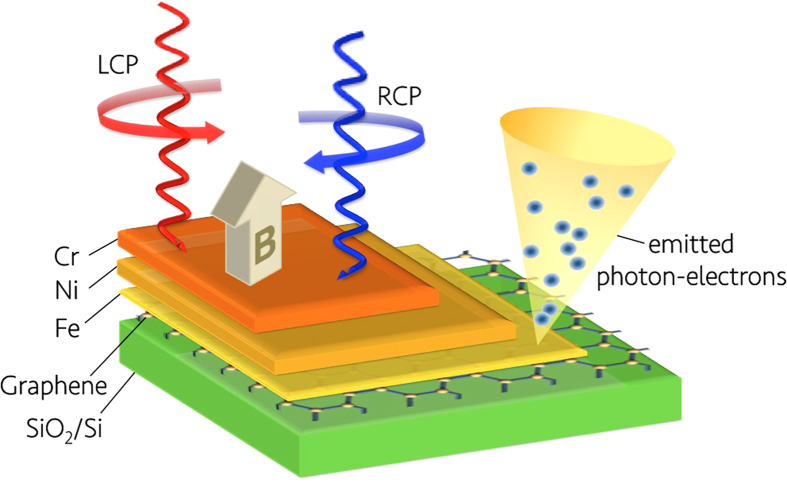


**Figure 2:**
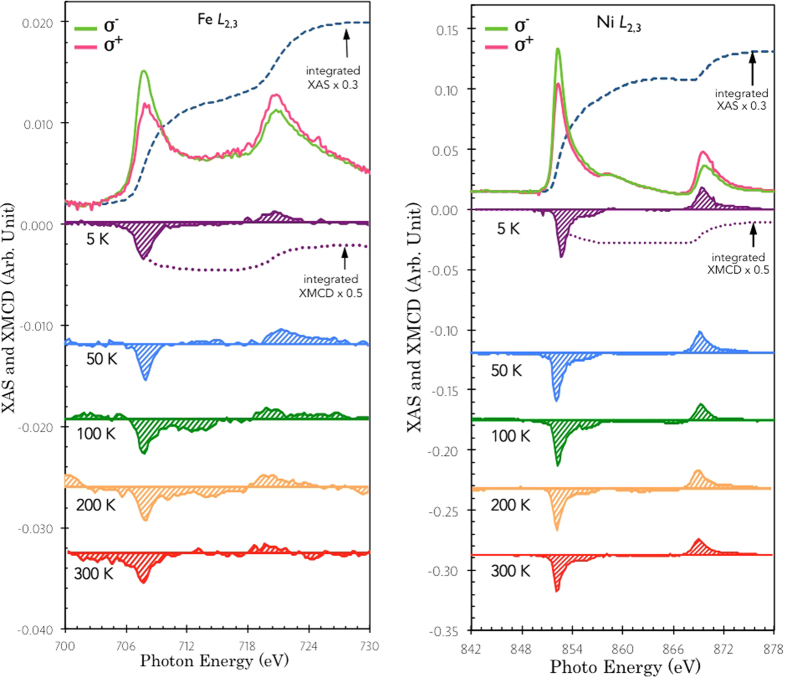
Typical pairs of XAS and XMCD spectra at 5–300 K of (**a**) the interfacial Fe and (**b**) the stabilizing Ni, respectively. The dashed lines represent the integrations of the spectra.

The XAS and XMCD of the Fe and Ni *L*_2,3_ absorption edges were performed on beamline I10 at the Diamond Light Source, UK. Circularly polarized X-rays with 100% degree of polarization were used in normal incidence with respect to the sample plane and parallel with the applied magnetic field, as shown in Fig. 1, in order to minimize the nonmagnetic asymmetries and saturation effects. The XAS spectra were obtained using both total electron yield (TEY) and total florescence yield (TFY) detection simultaneously. The XMCD was taken as the difference of the XAS spectra, i.e., σ^−^ − σ^+^, obtained by flipping the X-ray helicity at a fixed magnetic field of 3 T, under which the sample is fully magnetized with little paramagnetic contribution. The X-ray absorption (XAS) and XMCD of the Fe and Ni *L*_2,3_ absorption edges were performed and typical spectra obtained at 5–300 K are presented in Fig. 2a,b, respectively. The XAS spectra of the interface Fe and the stabilizing layer Ni for both left- and right-circularly polarized X-rays show a white line at each spin-orbit split core level without prominent splitting, suggesting that the sample has been well protected from oxidation.

**Figure 3:**
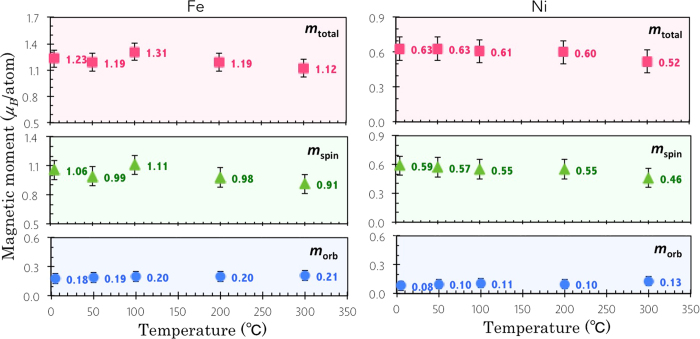


The spin (*m*_spin_) and orbital magnetic moment (*m*_orb_) were calculated by applying the sum rules^24^ to the integrated XMCD and summed XAS spectra of the Fe *L*_2,3_ edges based on
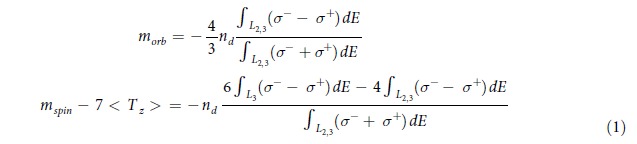
where the effective number of *3d*-band holes, *n*_h_, was assumed as 3.7 accounting for the charge transfer from Fe to the atop stabilizing Ni layer (*n*_h_ = 3.4 in bulk)^25^. In order to exclude non-magnetic parts of the XAS spectra, a sigmoidal function is used to fit the threshold^26^,^27^. As can be seen from Fig. 2a, both integrations (the dashed lines) of XMCD and summed XAS spectra become flat within the selected range, proving a proper background offset. The calculated *m*_spin_, *m*_orb_, and total magnetic moment (*m*_total_) of the interface Fe at temperatures from 5 K to 300 K were plotted in Fig. 3 (left). The *m*_total_ of Fe displays a decreasing trend with the increasing temperature from 1.23 *μ*_B_/atom at 5 K to 1.12 *μ*_B_/atom at 300 K, i.e., ~9% reduction, pointing to a *T*_C_ close to bulk-like Fe. At the lowest temperature (5 K), we obtained *m*_spin_ = (1.06 ± 0.1) *μ*_B_/atom, which is ~50% reduced from the bulk-like Fe (whose *m*_spin_ = 2.2 *μ*_B_/atom) and *m*_orb_ = (0.18 ± 0.02) *μ*_B_/atom, corresponding to an enhancement of ~200% compared to *m*_orb_ = 0.085 *μ*_B_/atom of that in the bulk^28^.

The enhancement of *m*_orb_ of Fe can be attributed to the symmetry breaking of the ultrathin films, in which the electrons are rather localized around the nucleus, leading to an orbital degeneracy lifting as that reported in Fe/GaAs^20^ and Fe/InAs^21^. A further factor of influence is the modified chemical environment of Fe due to the presence of graphene, as intensive spin and charge transfer can occur at the FM/graphene interface^25^,^29^,^30^,^31^. The stacking of the topmost FM with respect to graphene is another noteworthy question^32^. Within the context of the occupation sites, the best agreement between experiment and theory of the Fe/graphene heterojunction points to a coexistence of two types of domains, namely top-fcc and bridge-top domains^25^,^29^,^30^,^31^.

The calculated *m*_spin_, *m*_orb_, and *m*_total_ of the Ni stabilizing layer (see Fig. 3 (right)) are in good agreement with the previous reports of metallic Ni^33^. The sum-rules derived *m*_total_ of Ni exhibits a trend of slight decrease with the increasing temperature from (0.63 ± 0.06) *μ*_B_/atom at 5 K to (0.52 ± 0.06) *μ*_B_/atom at 300 K, pointing to a *T*_C_ close to the bulk-like Ni^34^. Unlike the interfacial Fe, whose *m*_orb_ shows a significant enhancement compared to that of bulk-like Fe, the Ni stabilizing layer shows a nearly quenched *m*_orb_. This is expected for bulk-like Ni, where the effect of the crystal field plays a dominant role. As an additional note, the graphene is likely to carry induced magnetic moment at the Fe/graphene interface^25^,^29^,^30^,^31^. However this magnetization is not sizable enough to quantitatively demonstrate with XMCD owing to the small crossection of C atoms.

### First principle simulations

In order to determine the electronic and magnetic ground state of the Fe ML on graphene, first it must be addressed where Fe likes to reside with respect to the C atoms. Periodic DFT calculations were performed to obtain the most energetically stable stacking of ML Fe on graphene. Three initial superstructures, namely, a Fe fcc(111) 1 × 1 primitive cell (2.55 × 2.55 Å^2^), a Fe bcc(100) 5 × 3 supercell (14.33 × 8.60 Å^2^), and a Fe bcc(110) 5 × 1 supercell (14.33 × 4.06 Å^2^), respectively, matching the graphene 6 × 2 supercell (14.82 × 8.56 Å^2^), a 6 × 1 supercell (14.82 × 4.28 Å^2^), and a 1 × 1 cell (2.47 × 2.47 Å^2^), are used to model Fe fcc(111), bcc(100) and bcc(110) MLs on graphene. For each superstructure, all atomic positions are relaxed until the atomic forces were smaller than 0.02 eV/Å. Figure S4 presents the initial (upper row) and the relaxed (lower row) geometries of Fe fcc(111), bcc(100) and bcc(110) stacking on graphene, respectively. The calculations suggest that the Fe prefers to follow the fcc(111)-like structure of the graphene substrate. Significant deformations were observed for both Fe bcc(110) and (100) on graphene due to the large lattice mismatch. The averaged Fe-Fe bond length after relaxation changes from 2.73 to 2.35 Å for Fe bcc(110), and from 2.91 to 2.38 Å for Fe bcc(100) ML, respectively, whilst that for Fe fcc(111) remains unchanged from 2.47 Å.

**Figure 4:**
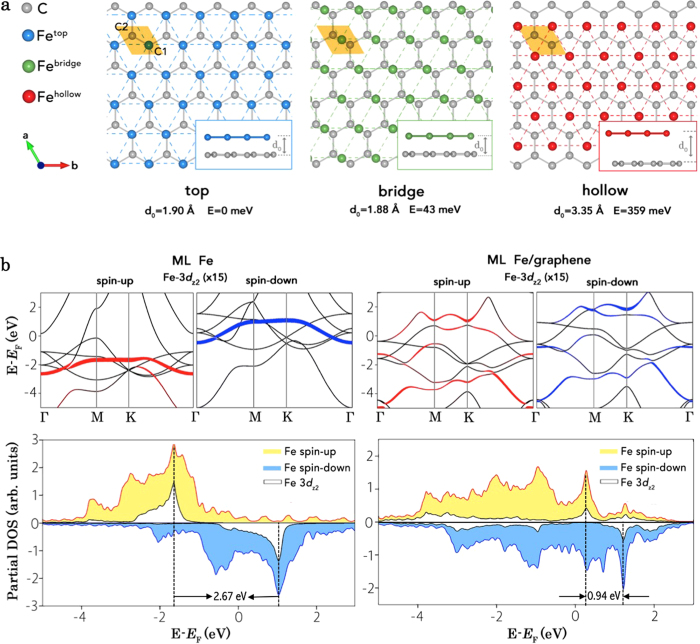
(**a**) Illustration of the three nonequivalent allocations of Fe on graphene, namely top (blue), bridge (green) and hollow (red), and their calculated equilibrium distance (d0) and system free energy (E), referenced to Fetop/graphene. The unit cell is shown as the gold-colored parallelogram. (**b**) Spin-resolved band structures for a freestanding Fe ML (left column) and the ML Fetop/graphene (right column), respectively, together with their corresponding partial DOSs.

It was found that the Fe atoms deposited on graphene prefer to follow fcc(111) stacking and there exist three inequivalent positions of the fcc Fe allocations on graphene as presented in Fig. 4a, namely, top (blue), hollow (red) and bridge (green), among which the top configuration is most energetically favorable with an averaged equilibrium distance of 1.90 Å. The calculated magnetic moments of Fe for the three different configurations as well as those derived from the experimental measurements were gathered in Table 1. Consistent with the experiment, the theoretical simulation also reveals a ~50% reduction in the magnetic moment of Fe. Although Fe^top^ was found the most stable geometry on graphene according to the calculation, the observed numeric results from XMCD, i.e., *m*_spin_ = (1.06 ± 0.1) *μ*_B_/atom, is more likely a mixture of Fe^top^ (*m*_spin_ = 1.23 *μ*_B_/atom) and Fe^bridge^ (*m*_spin_ = 0.67 *μ*_B_/atom), given the small energy difference (∆E = 43 meV) of their calculated total energies.

The DFT derived spin-resolved band structures for a freestanding Fe ML (left column) and the ML Fe^top^/graphene (right column), respectively, together with their corresponding partial density of states (DOSs) are presented in Fig. 4b. Remarkable exchange splitting (2.67 eV) for the spin-up and spin-down electrons of the freestanding Fe were observed from the partial DOSs and were traced back to those of the Fe 3*d*_*z2*_ orbitals (see the white shed areas), which is responsible for the large *m*_spin_ (2.76 *μ*_B_/atom). In contract, the Fe 3*d*_*z2*_ orbitals in ML Fe^top^/graphene become strongly delocalized in the reciprocal space and tend to show an *sp*-orbital-like partial band character. The exchange splitting sharply reduces from 2.67 eV to 0.94 eV due to the presence of graphene, pointing to a weakened exchange interaction between the 3*d* electrons of the Fe in Fe^top^/graphene and consequently a reduced spin polarization near the Fermi level (*E*_F_). Further examinations of the symmetry matching and spatial overlap suggest that the strong Fe-C hybridization predominantly originates from that between the C1 *p*_z_ orbitals and the Fe 3*d*_z2_ orbitals, although the interactions between the C2 *p*_z_ orbitals and the Fe 3*d*_xz_ and 3*d*_yz_ orbitals cannot be neglected (here, C1 stands for the C atom located underneath the Fe atom in Fe ML, while C2 is located at the hollow site, as shown in Fig. 4a).

**Figure 5:**
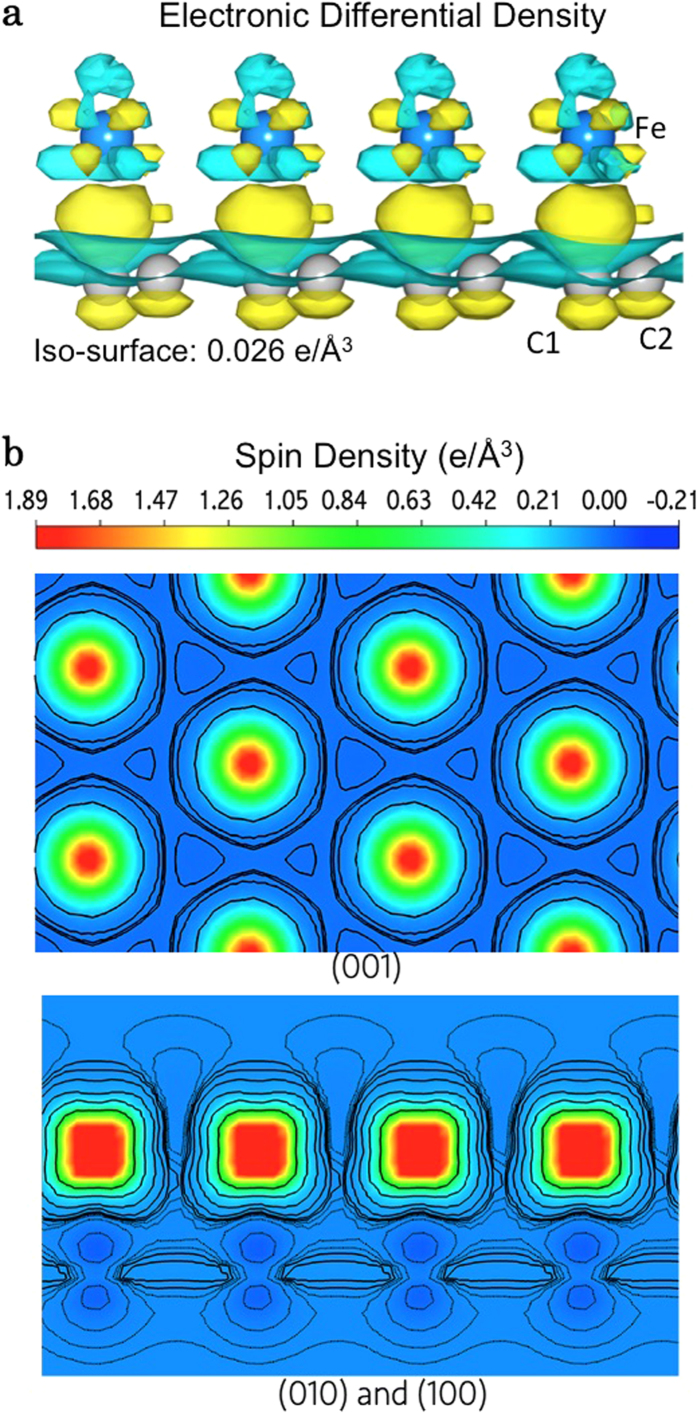
(**a**) 3D differential electronic density map, in which the yellow and green colored volumes represent the charge accumulation and depletion, respectively. The iso-surface corresponds to 0.026 e/Å3. (**b**) Spin polarization distribution in top ((001) direction) and side ((010) and (100) directions) views, respectively.

Figure 5 presents the differential electronic and spin density map of the Fe^top^/graphene. The 3D charge accumulation and depletion was obtained by subtracting the charge density of Fe^top^/graphene from that of the freestanding Fe ML and graphene as shown in Fig. 5a. The Bader charge analysis suggests that by average 0.13 *e* is transferred from Fe to C atoms per unit cell. This substantial charge transfer mainly occurs at the interfacial region of the Fe^top^/graphene and predominately via the C1 *p*_*z*_ orbitals. The Fe-C hybridization, in turn, slightly polarizes the C atoms in Fe^top^/graphene in opposite direction to the Fe moments (*m*_spin_ = −0.02 *μ*_B_/atom for the C1 and −0.01 *μ*_B_/atom for the C2 atoms) as can be seen from Fig. 5b, and consequently the Dirac point of intact graphene is destroyed. Further details of the induced magnetization of the C atoms can be found in the Supplementary Materials. Moreover, the spin and charge transfer of the metastable Fe^bridge^/graphene (not shown) are qualitatively same but quantitatively different as that of Fe^top^/graphene (not shown) and *m*_spin_ of −0.008 *μ*_B_/atom are induced for both the C1 and C2 atoms.

As an additional note, the effect of the topmost Ni was also considered. The bulk-like Ni atop the Fe ML serves as a stabilizing layer, in order to make the ML Fe in such configuration to be representative of the interface Fe of a bulk-like Fe on graphene. The two FM layers are expected to ferromagnetically couple with each other, provided an atomically clean interface is prepared, e.g., using UHV growth techniques. The calculation suggests that the stabilizing Ni layer has a limited influence on the underneath Fe by offering only a small charge transfer, i.e. 0.017 e/atom. In the fully relaxed structure, the Ni tends to pull the Fe slightly away from the graphene substrate, resulting in a higher *m*_spin_ of 1.53 μ_B_/atom for Fe. The inclusion of the topmost 7 MLs Ni has not modified the favorable stacking of the ML Fe in conjunction with the graphene. Furthermore, the calculated average *m*_spin_ of the 7 ML Ni atop Fe is 0.59 μ_B_/atom, well reproducing the experimental value of *m*_spin_ = (0.59 ± 0.06) *μ*_B_/atom obtained from XMCD.

## Conclusions

To summarize, we have demonstrated an unambiguous ferromagnetic epitaxial Fe/graphene interface by XMCD. This was aided by the inclusion of a Ni stabilizing layer atop the Fe film, which facilitated the study of the temperature dependence of the Fe/graphene interface. We observed strong dichroic XAS spectra of Fe, suggesting a well-maintained ferromagnetic order of the Fe^top^/graphene interface up to room temperatures. We obtained a reduced but still sizable magnetic moment of the ML Fe on graphene, i.e., (1.23 ± 0.1) *μ*_B_/atom, which is well resembled by the DFT calculations. The observed suppression of the Fe magnetization is attributed to the strong hybridization between the Fe 3*d*_*z2*_ and the C 2*p*_*z*_ orbitals, leading to greatly reduced exchange splitting, and the *sp*-orbital-like behavior of the Fe 3*d* electrons due to the presence of graphene. Our study restores a realistic scenario of the proposed graphene-based transistors and addresses the open question of the magnetic and electronic nature of the Fe/graphene interface. Although possibilities such as the discontinuous growth of the ML Fe and the contact of Fe with the SiO_2_ substrate cannot be fully excluded from the experiment, our study has demonstrated a valuable model of exploring the interfacial magnetism of FM on graphene and the results are encouraging based on the contemporary sample preparation technique. Future work to explore the tuning of the spin polarized band structure of both the FM and graphene via the interface engineering will be of great interest and have strong implications for both fundamental physics and the emerging spintronics technology.

## Methods

The single layer graphene used in this study was prepared by chemical vapor deposition (CVD) on Cu foil and then transferred on top of 300 nm SiO_2_/Si substrate^35^,^36^. After 1 hour of annealing at 200 °C, 1 ML Fe was grown on the graphene by molecular beam epitaxy (MBE) using an e-beam evaporator, whose rate was monitored by a quartz microbalance and calibrated by ex-situ atomic force microscope (AFM). 30 ML Ni was then deposited onto the Fe and the Ni/Fe/graphene was finally capped by 15 ML Cr for easy transport to the synchrotron facility. Raman scattering measurements were performed on the FM/graphene and an as-grown area of the sample without FM deposition. From both part the character spectra of graphene were obtained, suggesting that the structure of graphene was well maintained after the transfer^36^, annealing, and the FM deposition processes. Further details of the Raman spectroscopy experiment can be found in the supplementary materials.

The first-principle calculations were performed using the projector augmented wave methods implemented in the Vienna *ab initio* simulation package (VASP)^37^,^38^. The electron exchange and correlation interactions were described by the local density approximation (LDA)^39^. The plane wave kinetic energy cutoff was set to be 520 eV. A Monkhorst-Pack mesh^40^ of 31 × 31 × 1 *k*-points was used to sample the two-dimensional Brillouin zone for the thin film electronic structure calculations. The total energies were obtained using the tetrahedron method with the Blöchl corrections. In each unit cell, all atomic positions are fully relaxed with the conjugate gradient procedure until the residual forces vanished within 0.02 eV/Å. The vacuum space of 15 Å was set to separate the interactions between neighboring slabs of the unit cells. Test calculations have been performed for the bulk bcc Fe (*a*_0_ = 2.87 Å) and the freestanding fcc monolayer Fe (*a*_0_ = 2.55 Å) using this method and the results compare well with the pioneering reports^25^,^29^,^30^,^31^,^41^,^42^,^43^, proving the validity of the selected computational method^44^,^45^.

## Additional Information

**How to cite this article**: Liu, W. Q. *et al.* Atomic-Scale Interfacial Magnetism in Fe/Graphene Heterojunction. *Sci. Rep.* **5**, 11911; doi: 10.1038/srep11911 (2015).

## Supplementary Material

Supplementary Information

## Figures and Tables

**Table 1:** The experimentally measured (XMCD) and DFT calculated magnetic moments of Fe in various configurations.

**System**	**Ref.**	**Method**	**stacking**	***m*_spin_ (*μ*_B_/atom)**	***m*_orb_ (*μ*_B_/atom)**
Fe/graphene	[*]	XMCD		1.06 ± 0.1	0.18 ± 0.02
Fe^top^/graphene	[*]	DFT	fcc	1.23	
Fe^bridge^/graphene	[*]	DFT	fcc	0.67	
Fe^hollow^/graphene	[*]	DFT	fcc	2.57	
freestanding ML Fe	[*]	DFT	fcc	2.76	
Fe/InAs	21	XMCD	bcc	1.22 ± 0.12	0.22 ± 0.03
Fe/GaAs	20	XMCD	bcc	1.84 ± 0.21	0.25 ± 0.05
bulk-like Fe	[*]	DFT	bcc	2.15	
bulk-like Fe	28	XMCD	bcc	1.98	0.086

^*^refers to the results in the current work.

## References

[b1] Geim A. K. & Novoselov K. S. The rise of graphene. Nat. Mater. 6, 183–191 (2007). 17330084 10.1038/nmat1849

[b2] Min H. *et al.* Intrinsic and Rashba spin-orbit interactions in graphene sheets. Phys. Rev. B 74, 165310 (2006).

[b3] Tombros N., Jozsa C., Popinciuc M., Jonkman H. T. & van Wees B. J. Electronic spin transport and spin precession in single graphene layers at room temperature. Nature 448, 571–574 (2007). 17632544 10.1038/nature06037

[b4] Schwierz F. Graphene transistors. Nat. Nanotechnol. 5, 487–96 (2010). 20512128 10.1038/nnano.2010.89

[b5] Li X., Wang X., Zhang L., Lee S. & Dai H. Chemically derived, ultrasmooth graphene nanoribbon semiconductors. Science 319, 1229–1232 (2008). 18218865 10.1126/science.1150878

[b6] Wang X. *et al.* Room-Temperature All-Semiconducting Sub-10-nm Graphene Nanoribbon Field-Effect Transistors. Phys. Rev. Lett. 100, 206803 (2008). 18518566 10.1103/PhysRevLett.100.206803

[b7] Prinz G. A. Magnetoelectronics. Science 282, 1660–1663 (1998). 9831549 10.1126/science.282.5394.1660

[b8] Ohishi M., Shiraishi M., Nouchi R., Nozaki T., Shinjo T. & Suzuki Y. Spin Injection into a Graphene Thin Film at Room Temperature. Jpn. J. Appl. Phys. 46, L605–L607 (2007).

[b9] Iqbal M. Z. *et al.* Spin valve effect of NiFe/graphene/NiFe junctions. Nano Res. 6, 373–380 (2013).

[b10] Karpan V. *et al.* Graphite and graphene as perfect spin filters. Phys. Rev. Lett. 99, 176602 (2007). 17995355 10.1103/PhysRevLett.99.176602

[b11] Han W. & Kawakami R. K. Spin relaxation in single-layer and bilayer graphene. Phys. Rev. Lett. 107, 047207 (2011). 21867040 10.1103/PhysRevLett.107.047207

[b12] Novoselov K. S. *et al.* A Electric Field Effect in Atomically Thin Carbon Films. Science 306, 666–9 (2004). 15499015 10.1126/science.1102896

[b13] Ando K. & Saitoh E. Inverse spin-Hall effect in palladium at room temperature. J. Appl. Phys. 108, 113925 (2010).

[b14] van ’t Erve O. M. J., Friedman A. L., Cobas, E.,Li C. H., Robinson J. T. & Jonker B. T. Low-resistance spin injection into silicon using graphene tunnel barriers. Nat. Nanotechnol. 7, 737–742 (2012). 23023645 10.1038/nnano.2012.161

[b15] Sessi V. *et al.* Single 3 d transition metal atoms on multi-layer graphene systems: electronic configurations, bonding mechanisms and role of the substrate. New J. Phys. 16, 062001 (2014).

[b16] Johll H. & Kang H. C. Density functional theory study of Fe, Co, and Ni adatoms and dimers adsorbed on graphene. Phys. Rev. B 79, 245416 (2009).

[b17] Yang C.-K., Zhao J. & Lu J. Magnetism of transition-metal/carbon-nanotube hybrid structures. Phys. Rev. Lett. 90, 257203 (2003). 12857161 10.1103/PhysRevLett.90.257203

[b18] Yagi Y. *et al.* Stable geometries and magnetic properties of single-walled carbon nanotubes doped with 3*d* transition metals: A first-principles study. Phys. Rev. B 69, 075414 (2004).

[b19] Li J. *et al.* Magnetic dead layer at the interface between a Co film and the topological insulator Bi_2_Se_3_. Phys. Rev. B 86, 054430 (2012).

[b20] Claydon J., Xu Y., Tselepi M., Bland J. & van der Laan G. Direct observation of a bulklike spin moment at the Fe/GaAs(100)-4 × 6 interface. Phys. Rev. Lett. 93, 037206 (2004). 15323864 10.1103/PhysRevLett.93.037206

[b21] Xu Y. B. *et al.* Interface magnetic properties of epitaxial Fe-InAs heterostructures. IEEE Trans. Magn. 38, 2652–2654 (2002).

[b22] Brar V. W. *et al.* Gate-controlled ionization and screening of cobalt adatoms on a graphene surface. Nat. Phys. 7, 43–47 (2010).

[b23] Eelbo T. *et al.* Adatoms and Clusters of 3d Transition Metals on Graphene: Electronic and Magnetic Configurations. Phys. Rev. Lett. 110, (2013). 10.1103/PhysRevLett.110.136804 23581356

[b24] Thole B. T., Carra P., Sette F. & van der Laan G. X-Ray Circular dichroism as a probe of orbital magnetization. Phys. Rev. Lett. 68, 1943–1946 (1992). 10045260 10.1103/PhysRevLett.68.1943

[b25] Weser M., Voloshina E. N., Horn K. & Dedkov Y. S. Electronic structure and magnetic properties of the graphene/Fe/Ni111 intercalation-like system. Phys. Chem. Chem. Phys. 13, 7534–9 (2011). 21431119 10.1039/c1cp00014d

[b26] Liu W. Q. *et al.* Spin and orbital moments of nanoscale Fe_3_O_4_ epitaxial thin film on MgO/GaAs(100). Appl. Phys. Lett. 104, 142407 (2014).

[b27] Liu W. *et al.* Enhancing Magnetic Ordering in Cr-doped Bi_2_Se_3_ using High-*T*_C_ Ferrimagnetic Insulator. Nano Lett. 15, 764–769 (2014). 25533900 10.1021/nl504480g

[b28] Chen C. T. *et al.* Experimental confirmation of the X-Ray magnetic circular dichroism sum rules for iron and cobalt. Phys. Rev. Lett. 75, 152–155 (1995). 10059138 10.1103/PhysRevLett.75.152

[b29] Soares E. A. *et al.* Graphene-protected Fe layers atop Ni(111): Evidence for strong Fe-graphene interaction and structural bistability. Phys. Rev. B 88, 165410 (2013).

[b30] Dedkov Y.S, Fonin M, Rüdiger U, Laubschat C. Graphene-protected iron layer on Ni(111). Appl. Phys. Lett. 93, 022509 (2008).

[b31] Sun X., Pratt A. & Yamauchi Y. First-principles study of the structural and magnetic properties of graphene on a Fe/Ni(111) surface. J. Phys. D. Appl. Phys. 43, 385002 (2010).

[b32] Tian C. S. *et al.* Body-Centered-Cubic Ni and Its Magnetic Properties. Phys. Rev. Lett. 94, 137210 (2005). 15904031 10.1103/PhysRevLett.94.137210

[b33] Wu R. & Freeman A. J. Limitation of the magnetic-circular-dichroism spin sum rule for transition metals and importance of the magnetic dipole term. Phys. Rev. Lett. 73, 1994–1997 (1994). 10056941 10.1103/PhysRevLett.73.1994

[b34] Vogel J. & Sacchi M. Polarization and angular dependence of the L_2,3_ absorption edges in Ni(110). Phys. Rev. B 49, 3230–3234 (1994). 10.1103/physrevb.49.3230 10011183

[b35] Reina A. *et al.* Large area, few-layer graphene films on arbitrary substrates by chemical vapor deposition Nano Lett. 9, 30–35 (2009). 19046078 10.1021/nl801827v

[b36] Li X., Cai W., An J., Kim S., Nah J. & Yang D. Large-area synthesis of high-quality and uniform graphene films on copper foils. Science 324, 1312–1314 (2009). 19423775 10.1126/science.1171245

[b37] Blöchl P. E. Projector augmented-wave method. Phys. Rev. B 50, 17953–17979 (1994). 10.1103/physrevb.50.17953 9976227

[b38] Kresse G. & Furthmuller J. Efficient Iterative Schemes for Ab initio total-energy calculations using a plane-wave basis set. Phys. Rev. B 54, 11169–11186 (1996). 10.1103/physrevb.54.11169 9984901

[b39] Perdew J. P. & Zunger A. Self-interaction correction to density-functional approximations for many-electron systems. Phys. Rev. B 23, 5048–5079 (1981).

[b40] Monkhorst H. J. & Pack J. D. Special points for Brillouin-zone integrations. Phys. Rev. B, 13, 5188–5192 (1976).

[b41] Castro Neto A. H., Peres N. M. R., Novoselov K. S. & Geim a. K. The electronic properties of graphene. Rev. Mod. Phys. 81, 109–162 (2009).

[b42] Zhao J. *et al.* Free-standing single-atom-thick iron membranes suspended in graphene pores. Science 343, 1228–1232 (2014). 24626924 10.1126/science.1245273

[b43] Vinogradov N.A., Lundgren E. Vinogradov A. S. Mårtensson N.& Preobrajenski A. B. *et al.* Formation and structure of graphene waves on Fe(110). Phys. Rev. Lett. 109, 026101 (2012). 23030182 10.1103/PhysRevLett.109.026101

[b44] Heyd J., Scuseria G. E. & Ernzerhof M. Erratum: hybrid functionals based on a screened Coulomb potential. J. Chem. Phys. 124, 219906 (2006).

[b45] Perdew J. P., Burke K. & Ernzerhof M. Generalized gradient approximation made simple. Phys. Rev. Lett. 77, 3865–3868 (1996). 10062328 10.1103/PhysRevLett.77.3865

