# Supplementary material for: Atomic-Scale Interfacial Magnetism in Fe/Graphene Heterojunction
Source: Sci Rep. 2015 Jul 6;5:11911. doi: 10.1038/srep11911 (PMC4491707; doi:10.1038/srep11911)
Supplement: Supplementary Information [file srep11911-s1.pdf]

Supplementary Materials

Atomic-Scale Interfacial Magnetism in Fe/Graphene Heterojunction

W. Q. Liu,[1,2,†] W. Y. Wang,[3,4,†] J. J. Wang,[3,4] F. Q. Wang,[1] C. Lu,[2] F. Jin,[5] A. Zhang,[5] Q. M. Zhang,[5] G. van der Laan,[6] Y. B. Xu,[1,2,*] Q. X. Li,[3,4,*] R. Zhang[1,*]

[1]York-Nanjing Joint Centre for Spintronics and Nano Engineering (YNJC), School of Electronics Science and Engineering, Nanjing University, Nanjing 210093, China

[2] Spintronics and Nanodevice Laboratory, Department of Electronics, University of York, York YO10 5DD, UK

[3]Hefei National Laboratory for Physical Sciences at the Microscale, University of Science and Technology of China, Hefei 230026, China

[4] Synergetic Innovation Center of Quantum Information and Quantum Physics, University of Science and Technology of China, Hefei, Anhui 230026, China

[5] Department of Physics, Renmin University of China, Beijing 100872, China

[6] Diamond Light Source, Didcot OX11 0DE, UK

† Authors contribute equally to this paper.

* Electronic addresses: yongbing.xu@york.ac.uk, liqun@ustc.edu.cn, and rzhang@nju.edu.cn

1. Raman scattering measurement

The Raman scattering measurements were performed using a high-resolution monochromator (Jobin Yvon HR800) and 633 nm He-Ne laser in backscattering micro-configuration. The laser beam was focused into a spot on the sample surface with a diameter of ~5 μm and the beam power was kept below 1 mW to avoid heating effects. Figure S1 presents the Raman spectra collected on the FM/graphene and an as-grown area without FM deposition, which was obtained by shuttering half of the sample during the FM deposition. From both part similar line shape and peak width of the *D*- and *G*-bands were obtained, suggesting that the structure of graphene was well maintained after the transfer, annealing, and the FM deposition processes. The *G*-band peak position, which is a sensitive indicator of the

structural damage of graphene, shows no shift in the presence of the FM thin film, suggesting that the FM deposition has induced negligible defects into the graphene, if any. Meanwhile, the topmost FM induces a substantial shift of ~14 cm^{-1} at the peak position of the *D*-band, suggesting a considerable Fe-C interaction. As the intensity and shape of the *D*-band show little difference between those collected on graphene and FM/graphene, the observed Raman shift is more likely due to the energy exchange between Fe and vibrating C atoms rather than an impurity effect. According to the DFT calculations (see the main text), the electronic band dispersion of graphene can be modified via the hybridization with Fe and consequently shifts the *D*-band position by tuning the finite-*q* transferred hopping process.

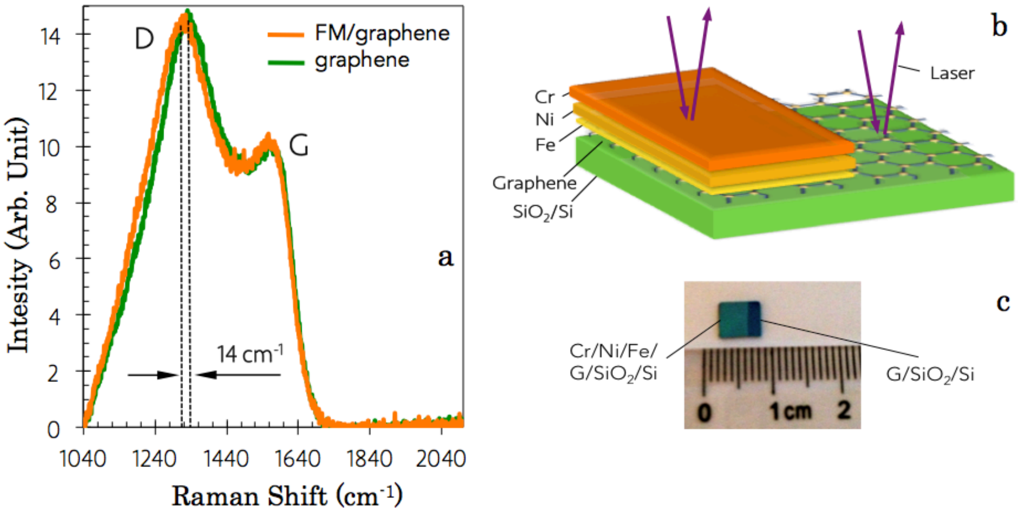

Figure S1 | Raman scattering measurement. (a) The Raman spectra collected on the FM/graphene and an as-grown area of graphene without FM deposition. The presence of topmost FM substantially induces a shift of ~14 cm^{-1} at the peak position of the *D*-band, suggesting that considerable Fe-C interactions have been involved. (b) Schematic diagram of the Raman scattering experimental configuration (c). Typical photograph of the FM/graphene sample used for the Raman scattering measurement. The boundary between the FM/graphene and graphene was created by shuttering half of the graphene substrate during the FM deposition. Here, G = graphene.

2. DFT simulations of the Fe stacking on graphene

Periodic DFT calculations were performed to obtain the most energetically stable stacking of ML Fe on graphene. Three initial superstructures, namely, a Fe fcc(111) 1 × 1 primitive cell (2.55 × 2.55 Å^2), a Fe bcc(100) 5 × 3 supercell (14.33 × 8.60 Å^2), and a Fe bcc(110) 5 × 1 supercell (14.33 × 4.06 Å^2), respectively, matching the graphene 6 × 2 supercell (14.82 × 8.56

Å^2), a 6 × 1 supercell (14.82 × 4.28 Å^2), and a 1 × 1 cell (2.47 × 2.47 Å^2), are used to model Fe fcc(111), bcc(100) and bcc(110) MLs on graphene. For each superstructure, all atomic positions are relaxed until the atomic forces were smaller than 0.02 eV/Å. Figure S2 presents the initial (upper row) and the relaxed (lower row) geometries of Fe fcc(111), bcc(100) and bcc(110) stacking on graphene, respectively. The calculations suggest that the Fe prefers to follow the fcc(111)-like structure of the graphene substrate. Significant deformations were observed for both Fe bcc(110) and (100) on graphene due to the large lattice mismatch. The averaged Fe-Fe bond length after relaxation changes from 2.73 to 2.35 Å for Fe bcc(110), and from 2.91 to 2.38 Å for Fe bcc(100) ML, respectively, whilst that for Fe fcc(111) remains unchanged from 2.47 Å.

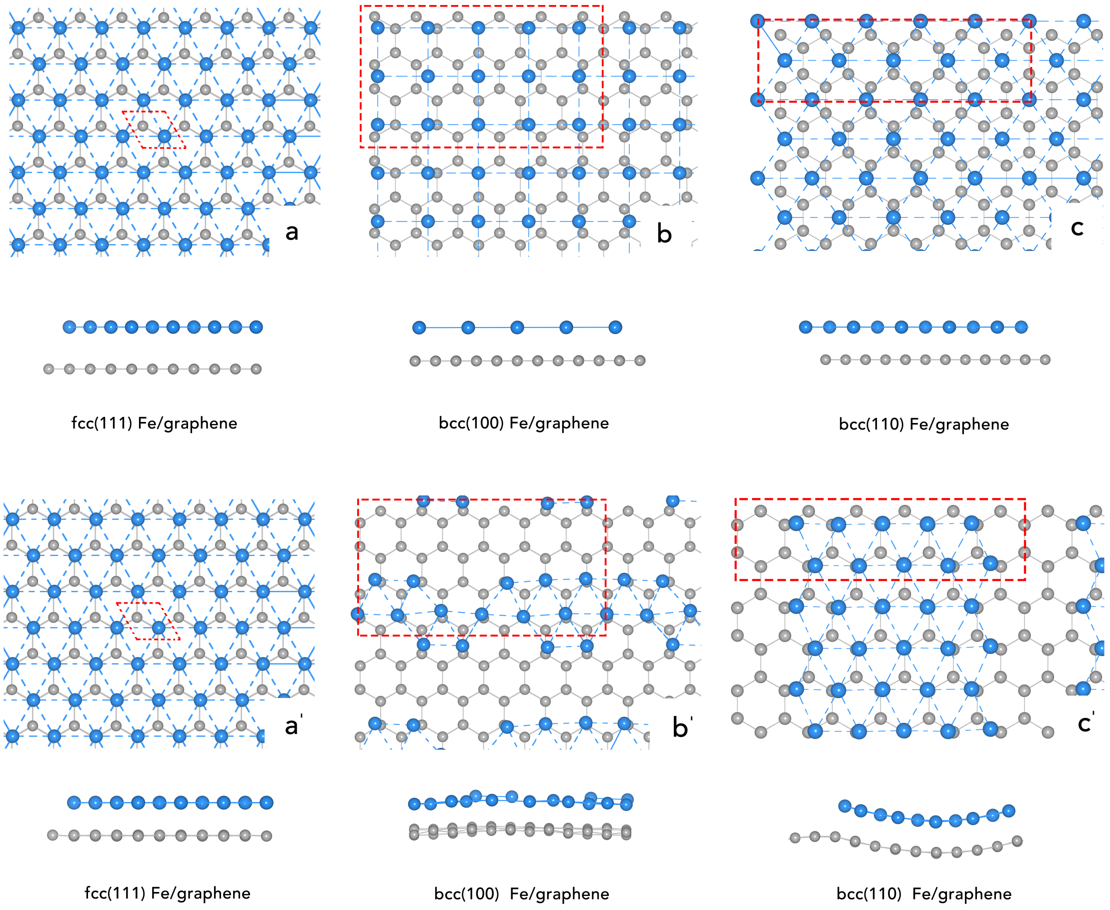

Figure S2 | The initial (upper row) and relaxed (lower row) geometries of Fe fcc(111), bcc(100) and bcc(110) stacking on graphene. The dashed rectangles indicate the supercells, which contains 3 atoms (2 C + 1 Fe atoms) for fcc(111), 63 atoms (48 C + 15 Fe atoms) for bcc(100), and 34 atoms (24 C + 10 Fe atoms) for bcc(110), respectively. Significant deformations were observed for both Fe bcc(110) and (100) on graphene due to the large lattice mismatch, whilst Fe fcc(111) retains its original symmetry.

3. The band structures of the C atoms in Fe/graphene

The DFT derived spin-resolved band structures for a freestanding graphene (upper row) and the ML Fe^{top}/graphene (middle and lower rows), respectively, together with their corresponding partial density of states (DOSs) are presented in Figure S3. Partial DOSs asymmetries are induced in graphene due to the presence of Fe and consequently the Dirac point of intact graphene, i.e., the cross point at valence band (VB) and conduction band (CB) at the K point, of intact graphene is destroyed. The Fe-C hybridization has stronger impacts on the C1 atoms than that on the C2 atoms because of the different symmetry matching and effective spatial overlapping. The subbands with C1 $2p_z$ and Fe $3d_{z2}$ partial band characters in Fe^{top}/graphene are both located at CB+1 and VB-3 while the subbands with C2 $2p_z$ character are mainly located at CB and VB-4, which correspond well only with these subbands with Fe $3d_{yz}$ character located at CB and VB-4.

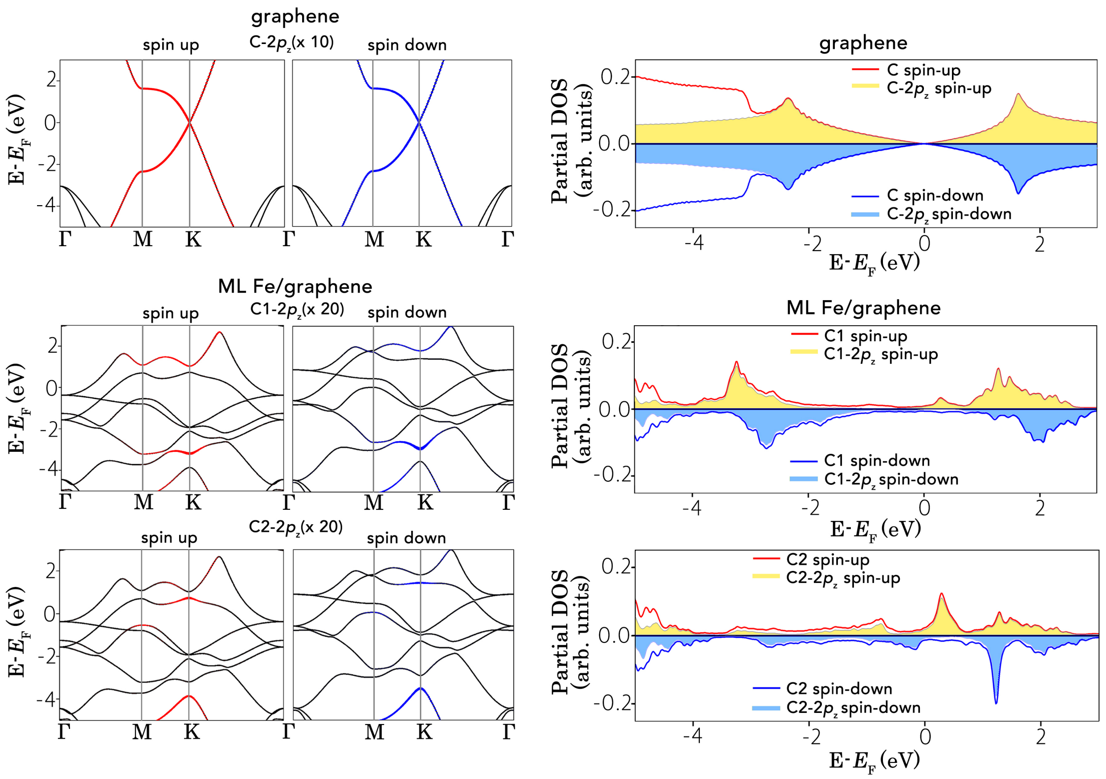

Figure S3 | The DFT derived spin-resolved band structures for a freestanding graphene (upper row) and the ML Fe^{top}/graphene (middle and lower rows), respectively, together with their corresponding partial DOSs. Partial DOSs asymmetries are induced in graphene due to the presence of Fe and consequently the Dirac point of intact graphene is destroyed.
